# Development of chitosan-coated agar-gelatin particles for probiotic delivery and targeted release in the gastrointestinal tract

**DOI:** 10.1007/s00253-020-10632-w

**Published:** 2020-05-06

**Authors:** Hanady A. Albadran, Andrea Monteagudo-Mera, Vitaliy V. Khutoryanskiy, Dimitris Charalampopoulos

**Affiliations:** 1 Department of Food and Nutritional Sciences, University of Reading, Whiteknights, PO Box 226, Reading, RG6 6AD UK; 2 Reading School of Pharmacy, University of Reading, Whiteknights, PO Box 224, Reading, RG6 6AD UK

**Keywords:** Agar-gelatin, Chitosan, Gel particles, Encapsulation, Probiotic, Faecal fermentation

## Abstract

This study reports the development of a novel and simple formulation for probiotic delivery using chitosan-coated agar-gelatin gel particles. This methodology involves the production of agar-gelatin particles by thermally treating a mixture of agar and gelatin solutions at high temperatures (121 °C) and subsequently coating with chitosan. The particles were able to protect the probiotic strain *Lactobacillus plantarum* NCIMB 8826 during incubation for 2 h in simulated gastric fluid (pH 2), as no statistically significant loss (*P* > 0.05) in cell concentration was observed, and also resist dissolution in simulated intestinal fluid (pH 7.2). Interestingly, this protection is related to the fact that the intense thermal treatment affected the physicochemical properties of agars and resulted in the formation of a strong and tight polymer network, as indicated by the X-ray diffraction (XRD) analysis. Using an in vitro faecal batch fermentation model simulating the conditions of the distal part of the large intestine (pH 6.7–6.9), it was demonstrated by quantitative real-time PCR that the majority of *L. plantarum* cells were released from the agar-gelatin particles within 30 to 48 h. Overall, this work led to the development of a novel methodology for the production of probiotic-containing particles, which is simpler compared with current encapsulation technologies and has a lot of potential to be used for the controlled release of probiotics and potentially other solid bioactives in the large intestine.

Key Points

• *Chitosan gel particles is a simple and scalable method of probiotic encapsulation.*

• *Autoclaving agar-gelatin particles increases their stability at low pH.*

• *Chitosan gel particles protected L. plantarum during gastrointestinal conditions.*

• *Probiotics could be controlled release in the colon using chitosan gel particles.*

**Electronic supplementary material:**

The online version of this article (10.1007/s00253-020-10632-w) contains supplementary material, which is available to authorized users.

## Introduction

Agar is a polysaccharide extracted from red seaweeds belonging to the genera of *Gelidium* spp. and *Gracilaria* spp. and consists of agarose (1-4)-linked 3,6-anhydro-a-L-galactose alternating with (1-3)-linked ß-D-galactose (Saxena et al. 2011). Agar has unique properties, i.e. it gels at 32–39 °C and melts at 85–95 °C, and has been used for preparing films either on its own (López de Lacey et al. 2014) or in combination with other materials such as milk protein (Letendre et al. 2002), shellac (Phan The et al. 2008), soy protein (Tian et al. 2011), and starch (Wu et al. 2009). Such films can have a variety of applications in the food, pharmaceutical, and cosmetic and personal care sectors. Agar has also been used to develop formulations for the delivery of bioactives in the gastrointestinal tract, more specifically to prepare tablets and microcapsules with gelatin (Lam et al. 2013).

Gelatin is a denatured protein that does not exist in nature, but it is derived from hydrolysed collagen extracted from the skin and bones of bovine or fish (Gómez-Guillén et al. 2002; Chen et al. 2014) and can be either positively charged if extracted with acid or negatively charged if extracted with alkaline (Duconseille et al. 2015). Gelatin forms thermally reversible gels with water and has a gel-melting temperature (~ 40 °C), depending on its concentration. The fact that the electrical and physical properties of gelatin can be altered depending on the processing operations has led to the development of gelatin-based controlled-release systems (Young et al. 2005). Gelatin is also widely used as a material for making hard and soft pharmaceutical capsules (Gullapalli and Mazzitelli 2017).

Probiotics are a group of bacteria described as ‘live microorganisms which when administered in adequate amounts confer a health benefit on the host’, with the most common commercial bacteria being of the genera *Bifidobacterium* or *Lactobacillus* (FAO/WHO 2002). The delivery of probiotics to the gut is often compromised because of their sensitivity to the low pH of the stomach and the high bile salt conditions of the small intestine (Cook et al. 2012). Encapsulation of probiotics into polysaccharide matrices, such as alginate, usually through an extrusion method, is a viable strategy for the oral delivery of probiotics, as it results in good protection; however, it is difficult to use in large-scale productions owing to the slow formation of the microbeads (Cook et al. 2012; Sarao and Arora 2017). Previously, it was established that probiotic bacteria, such as *Lactobacillus* and *Bifidobacterium* strains, encapsulated within calcium alginate beads coated with chitosan, may potentially survive the transit through the harsh environment of the stomach and release high levels of live probiotic in the small intestine (Cook et al. 2011; Cook et al. 2013b; Yeung et al. 2016; Yucel Falco et al. 2019). The protective effect of chitosan coating at high acidic conditions (pH < 3) was found to be due to its ability to delay acid diffusion into the microbeads, whereas the thickness and composition of the coating material influenced the release of the cells at pH > 6 (Cook et al. 2013b).

The aim of this work was to develop a novel, simple, and potentially scalable encapsulation method to produce chitosan-coated agar-gelatin particles containing probiotic bacteria, which are able to protect the cells in simulated gastric and small intestine conditions and release the cells in the large intestine. It is envisaged that such method could be used for better management of colonic disorders, such as irritable bowel syndrome and inflammatory bowel diseases.

## Materials and methods

### Bacterial strains and growth conditions

*Lactobacillus plantarum* NCIMB 8826 was obtained from the National Collection of Industrial and Marine Bacteria (NCIMB), UK. The bacterial strain was cultured at 37 °C for 16 h in Man Rogosa and Sharpe (MRS) broth at 200 rpm. Cells were harvested by centrifugation at 3200*g* for 15 min. The pellets were washed once using 0.1-M PBS and re-suspended in 10 mL of PBS, yielding a cell suspension with a concentration of around log_10_ 11.5 CFU/mL, determined by the spread plate method using MRS agar (2 days of incubation at 37 °C).

### Preparation and characterisation of bacterial loaded and unloaded agar-gelatin particles

Agar at different concentrations (1, 1.5, 3, 4, and 4.5% w/v) and gelatin (4% w/v) were dissolved separately in deionised water, at 70–80 °C for 2 h. The solutions were mixed at a ratio of 1:1, and the mixture was left to cool down to form a gel, or autoclaved at 121 °C for 15 min and then left to cool down. For the preparation of unloaded gel particles, 30 mL of the mixture (autoclaved or non-autoclaved) were poured onto a petri dish, left for 30 min at room temperature to solidify, and then cut into small particles of around 6 mm. In the case of bacterial loaded gel particles, these were prepared by mixing 1 mL of cell suspension with 9 mL of agar-gelatin mixture, and the above procedure followed. The initial cell concentration contained in 1 g of gel particles (approximately 10 particles) was around log_10_ 9.4 CFU/g.

Images of the unloaded gel particles were taken using a digital microscope (LEICA E Z4D), and processed using the ImageJ software to measure the size of the gel particles. Since the shape of the gel particles was regular, the size was determined using the average measurements of 3 diameters, produced from different batches of gel particles.

### Coating of agar-gelatin particles with chitosan

A chitosan solution (0.4% w/v) at pH 6.0 was prepared in 0.1-M acetic acid. The solution was filtered using a Whatman #4 filter paper, pasteurised at 72 °C for 30 s, and cooled down to room temperature. One gramme of loaded gel particles was added into 10 mL of chitosan solution, and the suspension was stirred for 40 min at 100 rpm. The gel particles were collected by filtration and washed with PBS before use. For bacteria enumeration, 1 g of gel particles was blended with 99 mL PBS in a stomacher (model 400 Circulation, Seward, UK) at 300 rpm for 20 min. The suspension was appropriately diluted, spread onto MRS agar plates, and incubated for 2 days at 37 °C. Bacterial colonies were counted and expressed as CFU/g.

### Viability of *L. plantarum* in agar-gelatin particles in simulated gastrointestinal fluids

Simulated gastric fluid (SGF) was prepared using 0.2% w/v NaCl and 0.3 g/L pepsin, adjusted to pH 2 by adding 1 M HCl. Simulated intestinal fluid (SIF) was prepared using 0.05 M of potassium phosphate buffer (KH_2_PO_4_) (pH 7.2) and 0.125 g/L pancreatic lipase. Both SGF and SIF were sterilised using a 0.2-μm Minisart microfilter (Sartorius Stedim Biotech, Germany). One gramme of gel particles was added to 9 mL of SGF, and the viability of bacteria was measured after incubation for 60 and 120 min at 37 °C. Subsequently, after 120 min in SGF, the gel particles were transferred to 9 mL of SIF, incubated at 37 °C, and the cell viability measured after 60, 120, and 180 min by the spread plate method using MRS agar.

### Faecal batch culture fermentation

Faecal batch culture fermentations were conducted to study the release of the probiotic in the colon environment. Glass sterile bioreactors (100 mL) were aseptically filled with 45 mL of basal medium consisting (per litre) of 2 g peptone water, 2 g yeast extract, 0.1 g NaCl, 0.04 g K_2_HPO_4_, 0.04 g KH_2_PO_4_, 0.01 g MgSO_4_·7H_2_O, 0.01 g CaCl·6H_2_O, 2-g NaHCO_3_, 2 mL Tween 80, 0.05 g hemin, 0.01 mL vitamin K_1_, 0.5 g/L-cysteine-HCl, 0.5 g bile salt, and 4 mL resazurin solution (0.25 g/L). Each vessel was inoculated with 1 g of agar-gelatin particles (approximately 10 particles) containing ~ 10^9^ CFU/g of *L. plantarum* and 5 mL of fresh faecal slurry in PBS, obtained by mixing 1 g of faecal sample from a healthy donor with 10 mL PBS and homogenisation in a stomacher (model 400 Circulation, Seward, UK) at 300 rpm for 2 min. Donors were healthy and had not received any antibiotic or probiotic treatment for at least 6 months prior to the experiment. Faecal samples were collected in sterile plastic containers which were stored in anaerobic jars containing AnaeroGen sachets (Oxoid, Basingstoke, UK). Stool samples were used within 2 h of collection.

The fermentation was conducted at 37 °C, controlled using a thermo-circulating water bath, under anaerobic conditions achieved by continuously passing nitrogen though the fermentation system. The pH was maintained in the range of 6.7–6.9 using a pH control system (Fermac 260, Electrolab, Tewkesbury, UK).

The fermentation was conducted in duplicate using faecal material from two different donors; a third run serving as the negative control was also conducted, using unloaded alginate-gelatin particles. During the fermentation, the dissolution of the agar-gelatin particles was monitored visually; samples were also collected from the reactor at 0, 8, 24, 48, and 72 h and centrifuged at 10,000 *g* for 5 min. The cell pellets were re-suspended in 50% glycerol-PBS and kept in − 20 °C prior to analysis.

### Extraction of DNA from faecal samples

Extraction of DNA from faecal batch culture samples was carried out according to Honda et al. (2011)). Briefly, the cell pellets collected as described in the previous section were washed with 1 mL PBS and re-suspended in 0.5 mL TES buffer (pH 8). Then, 8 μL lysozyme (10 mg/mL) and 2 μL mutanolysin (1 mg/mL) were added, and then the samples were incubated at 37 °C for 30 min. Subsequently, 10 μL proteinase K (20 mg/mL) and 10 μL RNase (10 mg/mL) were added and incubated at 65 °C for 1 h; 100 μL of 10% sodium dodecyl sulphate were then added, and the samples were incubated for a further 15 min at 65 °C. In a fume hood, 620 μL of phenol/chloroform was added, and the samples were gently mixed by inverting them, for about 2 min. The samples were then centrifuged at 6500*g* for 10 min. DNA was precipitated from the upper aqueous layer with ice-cold ethanol, then the samples were centrifuged at 13,000 *g* for 10 min. Supernatants were removed carefully, and the DNA dried before being eluted with 50 μL sterile water. The amount (ng/μL) of DNA was quantified by using a ND-1000 NanoDrop spectrophotometer.

### Quantitative real-time PCR

To quantify the levels of *L. plantarum* NCIMB 8826 and validate the release of the cells from the particles into the fermentation medium, primers targeting the plantaricin EF gene were used (plnEFfw 5′-CTA TTT CAG GTG GCG TTT TC-3′ and plnEFrev 5′-GTG GAT GAA TCC TCG GAC AG-3′) (Cho et al. 2010a). Plantaricin genes are known to occur only in *L. plantarum* strains, and thus plnEF primers have been used successfully to detect the plnEF gene in a number of studies (Cho et al. 2010b; Cho et al. 2011; Miller et al. 2011). Quantitative real-time polymerase chain reaction (qRT-PCR) was performed using a LightCycler® 480 system (Roche, USA). The reaction mixture (20 μL) contained 10 μL of IQ SYBR green PCR supermix (Bio-Rad), 2 μL of the DNA template, and 200 nM of each primer. qRT-PCR reactions were performed in triplicate under the following conditions: initial denaturation at 95 °C for 10 min, followed by 40 cycles of denaturation at 95 °C for 10 s, annealing at 54 °C for 30 s, and extension at 72 °C for 30 s. A melting curve analysis was done subsequently to determine the specificity of the PCR reaction by denaturing from 55 to 94 °C, immediately after the last cycle of each amplification. The PCR reaction efficiency (E = 10^1/-*S*^−1, where *S* = slope) was calculated from the log-linear part of a standard curve (Klein et al. 1999).

### X-ray diffraction analysis

Suspensions of solids made from 3% (w/v) agar, 4% (w/v) gelatin, and agar-gelatin 1:1 mixture (without cells) were analysed before and after autoclaving using an X-ray Bruker D8 Advance Powder diffractometer with copper source and wavelength of 1.54 °A. The samples were analysed between 2θ from 5 to 70°, with an angle size of 0.007° at 1 s per step. Data were processed using the Bruker EVA evaluation software package.

### Statistical analysis

The results are reported throughout as mean ± standard deviation. Statistical analysis of the data was conducted using ANOVA, version.17 of SPSS. *P* values < 0.05 were considered statistically significant.

## Results

### Development of agar-gelatin particles and evaluation of their solubility in simulated gastric fluid

Initial experiments investigated the effect of agar (4.5, 4, 3, 1.5, and 1% w/v) and gelatin (3% w/v) concentrations as well as autoclaving of the mixture at 121 °C for 15 min on the dissolution of the produced agar-gelatin particles after incubation in SGF for up to 120 min. In all cases, the gel particles (~ 6 mm) did not dissolve in SGF after 120 min, and based on the fact that with a higher agar concentration the particles had a more consistent spherical shape (Supplementary Fig. S1), 3% agar and 4% gelatin solution were selected for subsequent experiments, as this would most likely facilitate better coating with chitosan and thus enhance the stability of the probiotic.

**Fig. 1:**
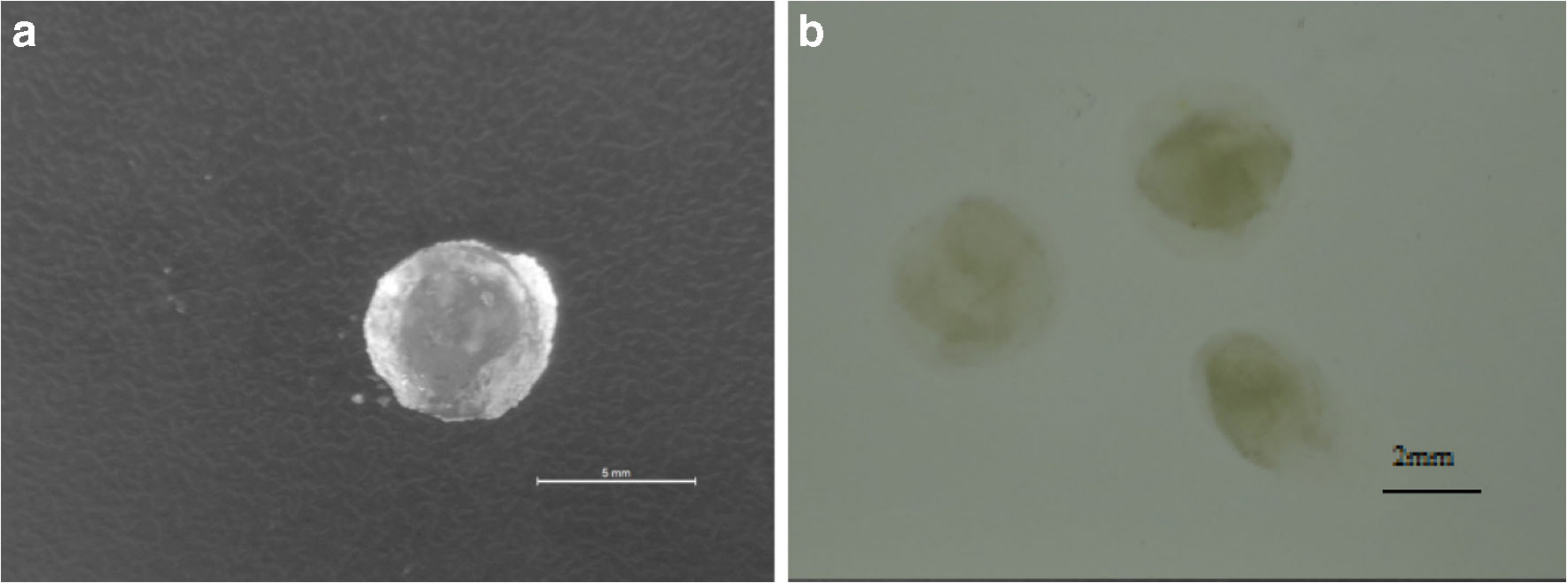
Images of agar-gelatin gel particles, prepared by mixing agar solution (3% w/v) with gelatin solution (4% w/v) at a ratio of 1:1, and then cooling the mixture at room temperature. **a** Gel particles. **b** Gel particles immediately after immersion in simulated gastric fluid (SGF). The scale shown in **a** is 5 mm, whereas in **b** is 2 mm

In order to evaluate the effect of autoclaving on the stability of the agar-gelatin particles in SGF, gel particles (~ 6 mm) were also produced by mixing 3% agar and 4% gelatin solutions without autoclaving the mixture in this case. Interestingly, upon immersing these particles in SGF, they disintegrated instantly as shown by the noticeable size decrease (Fig. 1), indicating that the autoclaving step positively influenced the stability of the agar-gelatin particles in SGF and prevented their dissolution.

### Viability of *L. plantarum* entrapped in uncoated and chitosan-coated agar-gelatin particles and release profile in simulated gastrointestinal fluids

**Fig. 2:**
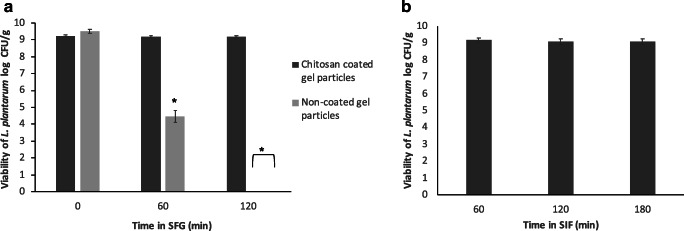
Viability of *L. plantarum* entrapped in uncoated and chitosan-coated gel particles, prepared by mixing and autoclaving 3% agar and 4% gelatin solutions, during incubation in simulated gastric fluid (SGF) for 2 h (**a**) and simulated intestinal fluid (SIF) for 3 h (**b**). Results are expressed as mean ± standard deviation (*n* = 3). No viable cells were detected in SGF after 2 h for uncoated gel particles (cell concentration < 2 log CFU/mL). * indicates significant difference (*P* ˂ 0.05) compared with the starting point (time 0)

Bacterial loaded agar-gelatin particles were produced and used as such or after coating with chitosan, in order to evaluate the cell viability and the release profile after exposure to SGF (up to 120 min) and subsequently to SIF (up to 180 min). In SGF (Fig. 2a), it can be observed that the gel particles without chitosan coating contained initially 9.50 ± 0.11 log CFU/g, whereas chitosan-coated particles contained 9.25 ± 0.07 log CFU/g. The viability of *L. plantarum* cells in uncoated gel particles decreased significantly (*P* < 0.05) (to ~ 4.5 log CFU/g) in SGF after 1 h, whereas after 2 h, no viable cells were detected in (< 2 log CFU/g). In contrast, the viability of the cells in chitosan-coated particles did not change significantly (*P* > 0.05) with time, and after 2 h, approximately 9.2 log CFU/g was present. Following 2-h exposure in SGF, the chitosan-coated gel particles were placed in SIF for 3 h (Fig. 2b), and the results showed that the cell viability did not change significantly (*P* > 0.05), and after 3 h, approximately 9.1 log CFU/g was present, indicating that the agar-gelatin particles did not disintegrate in SIF.

### XRD analysis

**Fig. 3:**
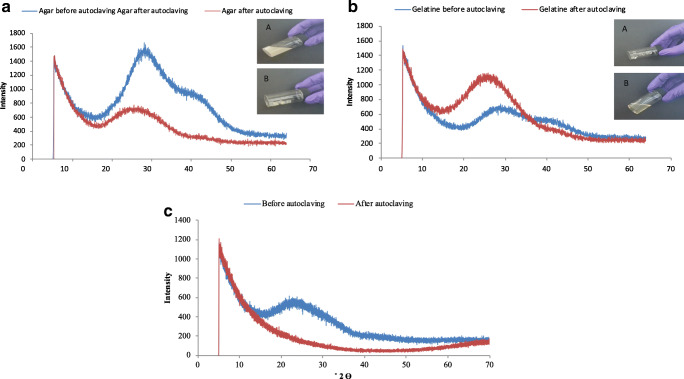
X-Ray diffraction patterns of **a** agar suspension (3% w/v) before and after autoclaving, **b** gelatin suspension (4% w/v) before and after autoclaving, and **c** agar-gelatin mixture (produced from mixing 3% w/v agar and 4% w/v gelatin at a ratio of 1:1) before and after autoclaving

X-ray diffraction (XRD) analysis of the 3% w/v agar solution and the 4% w/v gelatin solution and their 1:1 mixture was conducted both without autoclaving the solutions and after autoclaving at 121 °C for 15 min (Fig. 3). In the case of agar with no autoclaving (Fig. 3a), the agar gel gave a distinctive peak at 2θ = 27.9° and a secondary one at 2θ = 39.8°. After autoclaving, the predominant peak changed to 2θ = 23.7°, whereas the second peak disappeared. The insert photographs show that the agar without autoclaving was in a suspension form (semi-solid), whereas after autoclaving and cooling down (at room temperature for 30 min), it formed a gel. Gelatin without autoclaving exhibited two peaks of very low intensity (Fig. 3b), one at 2θ = 28.4° and a minor one at 39.8°, whereas after autoclaving, it exhibited only one peak, of considerably higher intensity than the non-autoclaved sample, at 2θ = 25.1°. The insert photographs show that the gelatin without autoclaving behaved as a gel. After autoclaving, the gelatin was cooled down to room temperature for 30 min and behaved as a liquid, whereas when cooled down to 4 °C, it behaved as a semi-solid.

When agar and gelatin were mixed together (Fig. 3c), the mixture without autoclaving consisted of a single peak at 2θ = 22.5° of relatively low intensity, whereas after autoclaving, no peak was detected. The insert pictures demonstrate that the mixture without autoclaving behaved as a semi-solid, whereas after autoclaving and cooling down, it behaved as a solid.

### Viability of *L. plantarum* entrapped in agar-gelatin particles and release profile in faecal batch culture fermentation

**Fig. 4:**
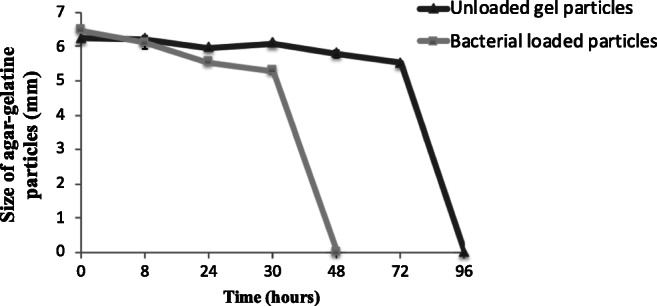
Changes in size of unloaded and *L. plantarum*-loaded particles during faecal batch culture fermentation. The particles were prepared by mixing agar solution (3% w/v) with gelatin solution (4% w/v) at a ratio of 1:1, and then cooling the mixture at room temperature

Unloaded and were added in a faecal batch fermentation system reflecting the physiological conditions of the distal part of the large intestine. The dissolution of the particles was monitored by visual observation, and the concentration of *L. plantarum* in the fermentation medium was quantified by using qRT-PCR. Figure 4 shows the changes in the size of the loaded and unloaded particles after 8, 24, 30, 48, 72, and 96 h. It can be observed that both loaded and unloaded particles dissolved during faecal fermentation, although the dissolution of the loaded particles was much faster (between 30 and 48 h) compared with the unloaded particles (72 to 96 h).

In order to verify the results from the visual observations, the changes in the concentration of total lactobacilli was measured by using qRT-PCR, using primers targeting the plantaricin EF gene, which is present in *L. plantarum*. To achieve quantification, a qRT-PCR standard curve relating viable cell counts to qRT-PCR signals was initially constructed using a pure *L. plantarum* culture. The correlation between the Ct (cycle threshold) value and the CFUs was highly linear; the slope of the qRT-PCR-generated standard curve equation was − 1.715 with a correlation coefficient of 0.99. The PCR reaction efficiency calculated from the log-linear part of the standard curve was 0.8. The amplification specificity of the qRT-PCR reaction with the plnEF primers was also investigated by using the melting curve analysis of the qRT-PCR products after the final amplification step. Using the melting curve analyses, no non-specific peaks could be detected in the reaction. In addition, no peak was obtained in the no-template control, indicating that neither primer pair dimers nor unspecific PCR products interfered with the qRT-PCR reaction (data not shown).

**Fig. 5:**
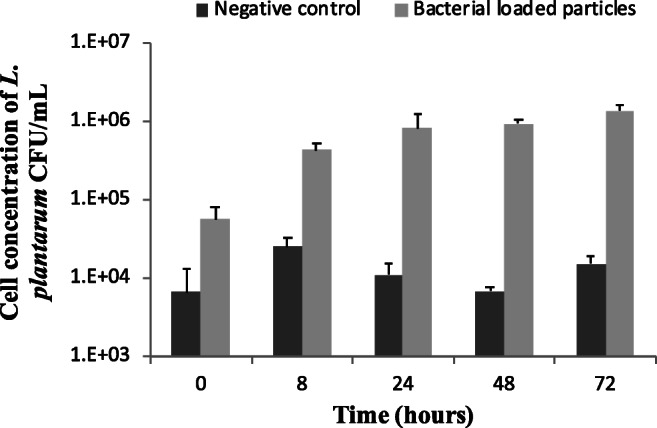
Cell concentration of *L. plantarum*, determined by using qRT-PCR, during batch culture faecal fermentation in the absence and presence of *L. plantarum*-loaded agar-gelatin particles (1 g of particles in 50 mL of fermentation culture). Error bars represent standard deviations from three replicate measurements

The results (Fig. 5) indicated that the majority of *L. plantarum* cells was released from the agar-gelatin particles within 48 h of incubation, with the initial cell concentration at time 0 h (immediately after immersion of the particles into the medium) being 5.6 × 10^4^ CFU/mL and increasing to 9.3 × 10^5^ and 1.3 × 10^6^ after 48 and 72 h, respectively. In the case of the negative control (faecal fermentation with no bacterial loaded particles), no significant changes were observed over the whole time course.

## Discussion

The study aimed to develop agar-gelatin particles, with and without coating with chitosan, containing probiotic bacteria to protect the cells in gastric conditions and release the cells in the large intestine rather than in the small intestine. The positive effect that autoclaving had on the stability of the produced agar-gelatin particles in SGF indicated the formation of a strong and tight polymer network. The intense thermal treatment of autoclaving compared with mild heat treatment affected the physicochemical properties of agar, as indicated by the XRD analysis. This was likely due to hydrogen bonding between the molecules inside the double-helical structure of agar that increases the strength of the gel, and therefore, a certain level of crystallisation could be present, as also demonstrated previously (Wu et al. 2009). On the other hand, autoclaving of gelatin most likely led to the decrease in OH^−^ bonding inside the triple-helical structure of gelatin, which caused its denaturation, thus producing an extremely weak gel (Brodsky and Ramshaw 1997), a hypothesis that is supported by the XRD results. The strong gel formed after autoclaving the mixture of agar-gelatin is reflected by the disappearance of all peaks in the diffraction graphs. Overall, these results are in agreement with previous research which has shown that a strong correlation generally exists between the strength of the gel and a diffraction pattern with no or low intensity peaks (Cheng et al. 2003; Zhai et al. 2004).

Although the agar-gelatin particles did not disintegrate in SGF, they did not protect the bacterial cells, indicating that acids were able to penetrate inside the core of the particles and kill the cells. The protection provided to the cells by chitosan coating on the agar-gelatin particles is attributed to the strong interaction between chitosan and gelatin, which takes place at pH 7, the pH of the chitosan solution. At pH 7, chitosan is positively charged and it interacts strongly with gelatin, which is negatively charged (Prata and Grosso 2015). To this end, Cheng et al. (2003) carried out X-ray diffraction analysis of mixtures of chitosan and gelatin at different concentrations and found that the crystallisation of the mixture decreased and the gelatinisation increased with increasing the amount of chitosan, as a result of the strong interactions between the NH_3_^+^ group in chitosan and the COO^−^ group in gelatin. Several researchers have demonstrated that chitosan coating of microcapsules, where the core material is another negatively charged polymer such as alginate, can protect probiotic cells in simulated gastric fluid (Krasaekoopt et al. 2004; Nualkaekul et al. 2012). In the mechanistic study by Cook et al. (2013a), a novel confocal laser-scanning microscopy (CLSM) method was developed for visualising pH changes within polymer matrices, which led to the generation of ‘pH maps’ showing the distribution of pH within the coated microcapsules. The maps revealed that the protection offered by the chitosan coating layer was due to a combination of buffering effect, which was seen to increase after coating with the basic chitosan, and an encroaching of low pH from the periphery of the microcapsule into the matrix rather than a bulk pH change, which was seen in the case of chitosan coating. It is likely that a similar mechanism was used in the case of the chitosan-coated agar-gelatin particles in SGF (pH 2), which would explain why the uncoated agar-gelatin particles did not protect sufficiently the cells, although confocal microscopy studies are needed to verify this.

The chitosan-coated agar-gelatin particles provided excellent protection to the cells during the 3-h exposure in SIF, which is of physiological relevance to an in vivo situation, and most interestingly, they did not disintegrate during this period. This is in contrast to what was observed in the case of coated and uncoated alginate microcapsules in previous research from our research group (Cook et al. 2011; Cook et al. 2013a), as well as other researchers (Kamalian et al. 2014; García-Ceja et al. 2015), where a fast release of the probiotic was observed in SIF. This is a new finding and could prove critical for targeting the release of bioactives, such as probiotic bacteria, in the large intestine and developing a novel delivery system using natural polymers. A number of approaches have been investigated to achieve the controlled release of probiotics targeting the colon. In a previous study from our research group, a layer-by-layer approach was developed for coating alginate microcapsules with chitosan, which resulted in the gradual release of the bacterial load in simulated small intestinal fluid over 240 min of exposure (Cook et al. 2013b). This approach could be modified, possibly using blends of coating materials and/or altering the number of coating layers, in order to minimise the dissolution of the microcapsule in the small intestine and ensure release of the load into the large intestine. However, such an approach would be difficult to scale up and commercialise due to the complexity of the layer-by-layer coating process. Another approach was the development of a multi-particulate dosage form to deliver a synbiotic (the combination of a probiotic microorganism and a prebiotic carbohydrate), consisting of poly(d,l-lactic-co-glycolic acid) (PLGA) microcapsules containing the prebiotic which were incorporated into an alginate–chitosan matrix containing the probiotic strain (Cook et al. 2014). The system was able to reduce the release of the probiotic in simulated small intestinal fluid and achieve significant release in simulated proximal colon (the early stage of the large intestine). However, from an industrial perspective, the production of such multi-particulate systems would be rather complicated. The method developed in this study is much simpler compared with the above methods and constitutes an entirely novel approach for the colonic delivery of probiotics to potentially address gastrointestinal disorders including antibiotic-associated diarrhoea, travellers’ diarrhoea, irritable bowel syndrome (IBS), irritable bowel disease (IBD), and Crohn’s disease.

Considering the results of the faecal batch fermentation experiment, it is likely that the dissolution of the particles observed (Fig. 4) could be due to prolonged incubation leading to the physical erosion of the particles. Moreover, it could be partly due to the activity of enzymes released by microbial constituents of the complex faecal microbiota, which are able to degrade the polymeric components of the particles, i.e. agar or gelatin. Agar can be degraded by agarolytic bacteria isolated primarily from marine environments (Michel and Czjzek 2013); however, there is little information regarding the potential agarolytic activities of human gut bacteria, which are likely to be low. However, a study in nature (Hehemann et al. 2010) demonstrated based on gut metagenome analyses that agarases and porphyranases (enzymes hydrolysing porphyrin, a sulphated carbohydrate derived from red algae) are frequent in the Japanese population and are absent in metagenome data from North American individuals. The authors suggested that as seaweeds make an important contribution to the daily diet in Japan, seaweeds with associated marine bacteria may have been the route by which these carbohydrases were acquired in human gut bacteria (Steck et al. 2011). On the other hand, Gram-positive commensal bacteria present in the gastrointestinal tract, in particular *Enterococcus faecalis*, have been shown to exert gelatinolytic activity (Steck et al. 2011). An additional interesting point is that with the available data, it is difficult to explain why the bacterial loaded particles dissolved faster than the unloaded ones (Fig. 5). One possible explanation could be that *L. plantarum* was able to slowly degrade the agar-gelatin particles; however, in general, lactobacilli do not have gelatinolytic and agarolytic activities; nevertheless, the enzymatic activities of this particular strain need to be studied to verify this. A more likely explanation is that the presence of entrapped bacterial cells within the agar-gelatin particles changed the structure of the particles, making the polymer matrix less strong, thus leading to faster dissolution. To this end, a more in-depth structural study of the agar-gelatin particles is needed to understand the dissolution pattern better.

The chitosan-coated agar-gelatin particles were able to protect the cells during incubation for 2 h in SGF and resist dissolution in SIF during 3 h of incubation, maintaining also cell viability. An interesting finding of this method used for preparing the particles was autoclaving the mixture of agar and gelatin at 121 °C for 15 min to influence on the particles’ dissolution properties, as the particles produced without autoclaving dissolved immediately in SGF. The chitosan-coated agar-gelatin particles had unique properties, and considering the simple method used for their production, they have a lot of potential to be used as novel formulation devices for the controlled release of probiotics and potentially other solid bioactives in the large intestine.

## Electronic supplementary material

ESM 1 (PDF 929 kb)
