# Supplementary material for: Development of chitosan-coated agar-gelatin particles for probiotic delivery and targeted release in the gastrointestinal tract
Source: Appl Microbiol Biotechnol. 2020 May 6;104(13):5749–57. doi: 10.1007/s00253-020-10632-w (PMC7306021; doi:10.1007/s00253-020-10632-w)
Supplement: Supplementary file 1 — (PDF 929 kb) [file 253_2020_10632_MOESM1_ESM.pdf]

Applied Microbiology and Biotechnology

Development of chitosan coated agar-gelatin particles for probiotic delivery and targeted release in the gastrointestinal tract

Hanady Albadran[a], Andrea Monteagudo-Mera*[a], Vitaliy Khutoryanskiy[b], Dimitris Charalampopoulos[a]

[a]Department of Food and Nutritional Sciences, University of Reading, Whiteknights, PO Box 226, Reading RG6 6AD, United Kingdom
[b]Reading School of Pharmacy, University of Reading, Whiteknights, PO Box 224, Reading RG6 6AD, United Kingdom

*Corresponding author: a.monteagudo@reading.ac.uk

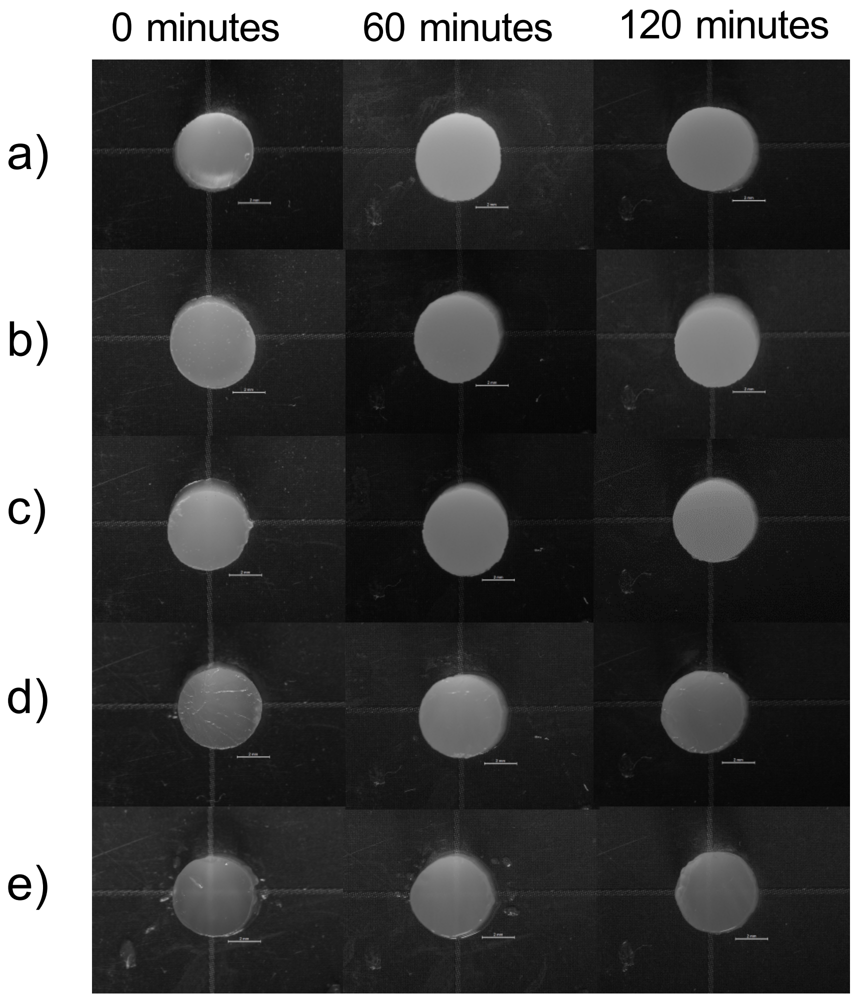

Supplementary Figure S1: Images of agar-gelatine gel particles in simulated gastric fluid (SGF) for up to 120 min of incubation. The gel particles were prepared by mixing various concentrations of agar solutions: a) 4.5, b) 4, c) 3, d)1.5 and e) 1 % w/v) with gelatine solution (3 %w/v) at a ratio of 1:1, and then autoclaving the mixture at 121 °C for 15 min and cooling to room temperature. The scale shown in all images is 2 mm.
